# Never miss a beep: Using mobile sensing to investigate (non-)compliance in experience sampling studies

**DOI:** 10.3758/s13428-023-02252-9

**Published:** 2023-11-06

**Authors:** Thomas Reiter, Ramona Schoedel

**Affiliations:** Department of Psychology, Ludwig-Maximilians-Universität München, Leopoldstraße 13, 80802 Munich, Germany

**Keywords:** Experience sampling, Ecological momentary assessment, ESM, Mobile sensing, Non-response, Compliance, Compliance bias

## Abstract

Given the increasing number of studies in various disciplines using experience sampling methods, it is important to examine compliance biases because related patterns of missing data could affect the validity of research findings. In the present study, a sample of 592 participants and more than 25,000 observations were used to examine whether participants responded to each specific questionnaire within an experience sampling framework. More than 400 variables from the three categories of person, behavior, and context, collected multi-methodologically via traditional surveys, experience sampling, and mobile sensing, served as predictors. When comparing different linear (logistic and elastic net regression) and non-linear (random forest) machine learning models, we found indication for compliance bias: response behavior was successfully predicted. Follow-up analyses revealed that study-related past behavior, such as previous average experience sampling questionnaire response rate, was most informative for predicting compliance, followed by physical context variables, such as being at home or at work. Based on our findings, we discuss implications for the design of experience sampling studies in applied research and future directions in methodological research addressing experience sampling methodology and missing data.

Trivial as it may sound, what most studies have in common is that they deal with data they have, not data they do not have. Missing data, however, can lead to problems or fallacies if not taken into account in a study’s design, data analysis, or interpretation of results (Graham, 2012; Little & Rubin, 1987). For example, individuals suffering from depression might be less willing to participate in a survey because of a lack of energy associated with their illness. The resulting systematic lack of data could lead to biased findings when estimating the prevalence of depression or its association with other variables of interest (Prince, 2012). Biased results, in turn, can have far-reaching consequences, for example, in informing policy makers to ensure adequate mental health care (Shorey et al., 2022).

Non-response bias is a challenge not only for traditional surveys but also for newer data collection approaches such as the experience sampling method (ESM) also often referred to as ecological momentary assessment or ambulatory assessment (Stone et al., 2023). First introduced by Larson and Csikszentmihalyi (1983), ESM has become a data collection tool widely applied across different disciplines, such as medicine, economics, computer science, and behavioral sciences. Its primary idea is to repeatedly assess individuals’ behavior, feelings, or thoughts on (pseudo-)random occasions in daily life. As the request to respond is often associated with some kind of audible signal, the repeatedly sent experience sampling (ES) questionnaires have historically often been called *beeps* (Csikszentmihalyi & LeFevre, 1989).

In survey research, the term *non-response (bias)* has become established to refer to missingness. To delineate the terminology used in ESM research, we follow scholars’ suggestion and use the term *(non-)compliance (bias)* (van Berkel et al., 2020). In doing so, we aim to highlight the repeated nature of assessments. We thereby also emphasize that we are considering the special case where participants initially committed to participate in a study but then failed to respond for a portion of a study’s ESM assessments. In contrast to one-time surveys, natural environments in which ESM studies are conducted come with even more reasons why participants might not answer specific beeps. The frequent need to answer ES questionnaires directly or in a timely manner adds the current context or what a person is doing as another momentary hurdle in complying with a specific beep (Stone et al., 2023). These daily hurdles are also reflected in the average non-compliance rates in ESM studies ranging between 10% to 30% (Wrzus & Neubauer, 2022). Although ESM has meanwhile established as a data collection tool in psychological research, there are still many open questions regarding the validity of the self-report measures, in particular with respect to potential (non-)missingness of the data. Our study aims to address this gap by using a multi-method approach to explore compliance in an ESM study with a comprehensive set of potential hurdles participants are faced with in their natural daily environments.

## Scenarios of missing data in ESM studies

Traditionally, methodological literature distinguishes three types of missing data: *missing completely at random* (MCAR), *missing at random* (MAR), and *not missing at random* (NMAR) (Little & Rubin, 1987; Thoemmes & Mohan, 2015).

MCAR means that the missing observations must be a true random sample of all observations. That is, the probability of missing an observation does not depend on any observed or unobserved variables (Little & Rubin, 1987). An exemplary scenario in ESM studies for this type of missingness would be if the app used for delivering the ES questionnaires sometimes randomly crashes, independent of any participant state (i.e., feelings, behaviors, thoughts) at the moment of crashing.

The less restrictive concept of MAR assumes that missingness depends only on the observed values and not on any unobserved values of the variables (Newman, 2014). An exemplary scenario in ESM studies would be if older participants were more likely not to respond to ES questionnaires but the age of all participants is assessed (e.g., in a pre-questionnaire) and missingness does not depend on any other variables that were not assessed, after accounting for participants’ age (e.g., by including it as a control or auxiliary variable^1^).

Finally, the concept of NMAR assumes that missingness depends on the values of the missing variables themselves (Schafer & Graham, 2002). This is the case when the probability of missing an observation depends on variables that were not observed in the data set or on the values of the missing variables themselves (van Ginkel et al., 2007). An exemplary scenario in ESM studies would be if participants systematically fail to answer a mood questionnaire when they are in a specific situation, such as being "in the midst of a marital dispute" (Stone et al. , 2023, p. 15) , waiting for the dentist (which is probably unknown to the researcher), or, more general, whenever they are in a certain mood, for example, in a bad mood.

For the first two types of missingness, the methodological literature proposes easy-to-handle solutions for data analysis: MCAR observations can simply be neglected as listwise deletion will not introduce bias after data exclusion (although statistical power will decrease) (Allison, 2001). For MAR observations, well-known methods such as maximum likelihood estimation or multiple imputation can be used if researchers control for the variables that cause the missingness, even if this procedure does not explicitly model the process of missingness (Gelman & Hill, 2006; Mohan & Pearl, 2021). In contrast, NMAR observations come with certain (statistical) biases in the data analysis if the missingness is not explicitly modeled and, consequently, researchers are in danger of drawing wrong conclusions from their results (Gelman & Hill, 2006). To illustrate, imagine the following thought experiment: We conduct an ES study to examine the relationship between mood and the quality of social interactions. However, we do not consider the characteristics of the contexts our participants encounter when answering the ES questionnaires. Based on our study, we could reach null findings and conclude that mood is not related to the quality of social interaction. However, one explanation for the null findings could simply be that participants systematically did not respond to the beeps in certain contexts associated with bad mood (e.g., in a dentist’s waiting room), so we cannot detect a significant association because of the low variance in mood ratings. Thus, the conclusion that mood is unrelated to the quality of social interaction would be biased because we did not take into account that mood depends on contextual characteristics, that is, data were missing systematically. Thus, we cannot consider missing values as MAR if we systematically omitted mood reports in certain contexts, for example, if participants’ mood was exceptionally bad or good. Another example for NMAR in an ESM scenario is presented by Stone and Shiffman (2002): Investigating the interplay of chronic pain and psychological well-being, it seems natural due to the nature of the studied constructs that participants are less likely to answer ES questionnaires while experiencing pain, which could in turn introduce bias to the estimated association between pain and well-being. To conclude, especially data NMAR might be associated with problems and fallacies in ESM research.

In summary, there are different plausible scenarios for missing data but little is known about whether they should be considered MCAR, MAR, or NMAR in the ESM setting. Insight into these mechanisms associated with missing data would help researchers to avoid introducing bias in their statistical analyses. Important countermeasures such as controlling for variables that affect the probability of missingness or, when applying multiple imputation, including these variables in the imputation model are already available in the statistical literature (Gelman & Hill, 2006; Graham, 2009). To use them sensibly, an important next step is to investigate the nature of missingness in ESM studies.

### Sampling biases in ESM studies

Methodological ESM research has long recognized the importance of understanding missing data and scholars have started to investigate sampling biases in ESM studies from different perspectives.

#### Compliance at the study level

Most previous studies have examined participants’ overall compliance rate, that is, the percentage of beeps answered, and its association with both study and person characteristics. Study characteristics have ranged from more general design elements such as the overall study duration to more specific ones such as the implementation of particular ESM sampling schemes, the number and duration of ES questionnaires and thus participant burden, or compensation incentives; person characteristics have ranged from participants’ socio-demographic to psychological traits such as Big Five personality; (Ottenstein & Werner, 2021; Eisele et al., 2022; Hasselhorn et al., 2021; Harari et al., 2017; van Berkel et al., 2019; Vachon et al., 2019; Courvoisier et al., 2012). In more detail, some studies found negative associations between the overall compliance rate and ES questionnaire duration (Eisele et al., 2022), the number of study days, and the overall number of ES questionnaires (Ottenstein & Werner, 2021). In contrast, the implementation of specific sampling schemes (van Berkel et al., 2019) and monetary incentives (Harari et al., 2017) were found to be associated with higher compliance rates.

Overall the effects of study characteristics on compliance rate seem small or even negligible. Neither Wrzus & Neubauer (2022) nor a recent study by Hasselhorn et al. (2021) which experimentally manipulated different study characteristics found any effects of study characteristics on the overall compliance rate in ESM studies, except for incentivization. Moreover, design choices such as the number or duration of beeps are often determined by the research question at hand and thus cannot be easily adapted. Therefore, in our study, we consider study characteristics fixed and instead focus on person characteristics.

Gender has repeatedly been found to be related to overall compliance rate in previous studies. At the person level, the compliance rate was lower for male participants, and at the sample level, the compliance rate was lower for samples with a higher proportion of male participants (Silvia et al., 2013; Rintala et al., 2019). These effects, however, could not be replicated consistently (Howard & Lamb, 2023).

Depending on the field of ESM research, more specific person characteristics have been investigated in terms of overall compliance rate. For example, psychotic disorders were found to be related to decreased compliance (Vachon et al., 2019; Sokolovsky et al., 2014). In contrast, personality traits, for which an association with non-response has been hinted at by previous survey research (Rogelberg & Luong, 1998; Rogelberg et al., 2003; Satherley et al., 2015), have not been found to be associated to compliance in ESM studies (Courvoisier et al., 2012; Sun et al., 2020).

#### Compliance at the beep level

Few previous studies addressed participants’ compliance rate at the beep level by modeling the probability of participants answering specific beeps.

Some researchers have explored the association between the compliance rate at the beep level and context-related characteristics. The selection of contextual characteristics under study has ranged from easily accessible smartphone data like battery or charging status (van Berkel et al., 2020) and physical activity features (McLean et al., 2017) parameters of psychological context determined from the responses to the previous beep (e.g., participants’ mood or stress at the preceding beep)(Sokolovsky et al., 2014; Murray et al., 2023). In addition, electronically activated recorders (EARs) have been used to collect audio snippets of participants’ surroundings during beeps to infer the current context of participants (Sun et al., 2020). The inclusion of contextual characteristics such as physical activity or audio indicators captured via EAR (e.g., whether participants were engaged in social interaction at the time of the beep) increased the overall accuracy for predicting compliance rate at the beep level by 0.5 to 2 percentage points (McLean et al., 2017; Sun et al., 2020).

Apart from these contextual characteristics, some studies have focused on participants’ behavioral characteristics (Rintala et al., 2020; Sokolovsky et al., 2014). For example, if participants failed to answer the previous beep, they were more likely to miss the next beep (Rintala et al., 2020). Poly-substance users (i.e., participants using an illicit drug different to cannabis) were also more likely to miss a specific beep (Messiah et al., 2011). No effects on compliance rate at the beep level were, however, found for more general behaviors such as cigarette consumption during the last 30 days (Sokolovsky et al., 2014; Schüz et al., 2013) or aggressive behavior assessed at the previous beep (Murray et al., 2023). This partial absence of effects of behavioral characteristics could be a type of methodological artifact: Studies have frequently used only delayed behavioral information from the previous beep as predictors for compliance at the respective beep but not information on behavior at the beep itself because this information is missing if participants fail to answer the respective beep (e.g., Murray et al. , 2023; Rintala et al. , 2020; Silvia et al. , 2013). Nonetheless, information about behavior at the moment of the beep response itself could be an additional important source for predicting compliance at the beep level.

### How to collect data when they are missing

Previous findings paint a mixed picture of characteristics associated with compliance in ESM studies. Effects and conclusions for person and behavior characteristics are small and often not consistent across studies (Wrzus & Neubauer, 2022; Stone et al., 2023). In addition, the previous literature suggests that context characteristics add little to predicting compliance at the beep level (McLean et al., 2017; Sun et al., 2020). With a good portion of optimism, this could be considered good news for ESM researchers. If no characteristics are found to be systematically related to compliance, in the most optimistic interpretation, this could mean that missing data in ESM studies are simply missing completely at random. However, another explanation for the unclear pattern of findings could be the limited methodological scope of previous research. For example, some studies have used analogous and rather easy-to-backdate ESM methodology such as paper and pencil or call-based sampling (e.g., Rintala et al. , 2020; Courvoisier et al. , 2012), specific samples (e.g., 9th and 10th grade smokers, Sokolovsky et al. , 2014), or small sample sizes (e.g., n = 57, van Berkel et al. , 2019). When trying to detect associations between context or behavior characteristics and compliance, this methodological scope might have led to problems with detecting effects. While person characteristics are often collected via surveys once at the beginning of a study, information on context and behavior is missing – by definition of missing data – when participants do not respond to a specific beep, that is, when they do not provide a self-report on their current context and behavior via the respective ES questionnaire.

To overcome this methodological hurdle, one promising approach is mobile sensing that provides passively collected information (Harari et al., 2016). With smartphones being omnipresent in our daily lives, they are not only perfectly suited to supersede devices previously used for sending beeps in ESM studies such as paper-and-pencil diaries or personal digital assistants (e.g., PALM). They also offer the possibility of continuously collecting a variety of data types without the active engagement or interruption of participants’ day-to-day behavior, which in turn can be used to derive contextual and behavioral information, even if participants miss certain beeps (Harari et al., 2016; Elmer et al., 2022; Schoedel et al., 2023). Accordingly, scholars have recently pointed out the huge potential of using mobile sensing as a toolbox to gather further insight into compliance in ESM studies (Murray et al., 2023; Sun et al., 2020), for example, by using GPS data instead of self-reported information on locations (Sokolovsky et al., 2014).

### The present study

In this exploratory study, we adopt a multi-methodological approach and combine smartphone-based ESM with mobile sensing to investigate compliance and to obtain insight into the nature of missing data in ESM studies. In doing so, we address two research questions. In a first step, we investigate whether there are any characteristics at all that are systematically associated with compliance in ESM studies. If so, in a second step, we investigate which characteristics these are.

Therefore, our study focuses on two aspects that we think represent gaps in the current literature on compliance in ESM studies: First, most previous research has investigated overall but not beep level compliance. We think that zooming in on the beep level is an important next step to better understand overall compliance. What exactly makes participants miss a beep? Using information exclusively related to specific beeps might help us discover the (opposing) interplay of both more general participant characteristics and very specific contextual characteristics that might mask each other, finally leading to an inconsistent pattern or null findings at the overall level. Accordingly, our study focuses on compliance at the beep level. For this purpose, we use a large sample with 26,750 beeps sent to 592 participants collected across 10,856 days in total.

Second, many previous studies have focused on a small number of selected variables associated with compliance. That means that only person (Murray et al., 2023) *or* only contextual (Boukhechba et al., 2018) *or* only behavioral characteristics (Sun et al., 2020) *or* non-extensive and incomplete combinations thereof (Courvoisier et al., 2012) have been of specific interest. But, in order to better understand compliance in ESM studies, a *comprehensive combination* of these different categories of characteristics is still pending. Thus, in our study, we use an integrative approach combining 402 variables from all three categories (person, context, and behavior) to examine compliance in ESM studies.

## Method

The data for this study were collected in the Smartphone Sensing Panel Study (SSPS), an interdisciplinary research project at LMU Munich in cooperation with the Leibniz Institute for Psychology (ZPID; see Schoedel & Oldemeier , 2020). All procedures adhered to the General Data Protection Regulation (EU-GDPR) and received ethical approval. Not to go beyond the scope of this article, we focus our report on the procedures and measures relevant to our specific research question. A detailed description of the SSPS can be found in Schoedel & Oldemeier (2020).

### Transparency and openness

The study protocol of the SSPS was preregistered^2^. Due to the complexity of mobile sensing data and associated variable extraction procedures, our study adopts a purely exploratory perspective. Accordingly, we did not preregister our study but describe our approach transparently and in detail herein. Due to the privacy-sensitive nature of the mobile sensing data (e.g., timestamped logs in combination with GPS coordinates collected in daily life), we share our data set only as aggregated variables. However, we provide our preprocessing code, analysis code, and further supplemental material in our OSF repository^3^ to make our complete data handling pipeline transparent. Data preprocessing and analyses were conducted using the statistical software R (version 4.1.0, R Core Team , 2022). For reproducibility of our analysis, we used the package management tool renv() (Ushey, 2021) and provide a complete list of all R packages used in this paper in the renv.lock file in the OSF repository.

## Procedures

The initial sample of 850 participants from across Germany was collected with the help of a non-probability online panel provider according to quota representing the German population in terms of gender, age, education, income, religious denomination, and relationship status. In addition, participants had to be between 18 and 65 years of age, fluent in German, and for technical reasons be the sole user of a smartphone with Android version 5 or higher (see Schoedel & Oldemeier , 2020). Participants were compensated dependent on the number of study parts completed. After recruitment, participants were randomly assigned to one of two groups with a study duration of either three months ($n_{group1}$ = 191) or six months ($n_{group2}$ = 659).

Data collection started for all participants in May 2020. Participants were asked to install our self-developed Android-based mobile sensing app, called PhoneStudy^4^, on their private smartphone for the respective study duration. Using the app, various data types (phone usage, Bluetooth connectivity, GPS, etc.) were continuously collected in the background of the device. Each month, participants were sent a link to a 30-minute online survey via the app. These online surveys included questionnaires on socio-demographic and psychological measures (for a complete overview of included measures, see Schoedel & Oldemeier 2020).

The SSPS also included two 14-day ES waves (in July/August and September/October 2020) during which participants were asked to complete five-minute questionnaires on up to four occasions per day. The ES schedule was pseudo-randomized: Each day (from 7 a.m. to 10 p.m. on weekdays and from 9 a.m. to 11 p.m. on weekends) was divided into four equally sized time windows and two to four of these time windows were randomly chosen to schedule one ES questionnaire. The timing of a beep within a time window was again randomly chosen while maintaining a minimum interval of 60 minutes between two consecutive beeps. Participants were informed about the ES questionnaire via a notification as soon as they actively used their smartphone for the first time after the scheduled time for the respective beep. Accordingly, the time at which a beep was scheduled did not necessarily match the time at which the notification was presented on the participant’s screen. If a participant did not use the smartphone in a time window in which a beep was scheduled, the beep was overwritten and thus the participant did not receive it. This procedure was chosen because our study design required a careful trade-off between sending ES questionnaires randomly but not provoking artificial smartphone usage and thus not distorting or interrupting participants’ natural behavior (van Berkel et al., 2019).

### Sample

For data quality reasons, we applied several exclusion criteria. For example, we excluded participants who decided to cancel their participation within the first day of the panel study or who had technical problems. As our central research focus was compliance in ESM studies at the beep level, we excluded participants if their study behavior suggested that they were not seriously taking part in the ES waves. This was particularly important to check, as individual study parts such as online surveys, ES, and mobile sensing were compensated independently of each other, and participants were not generally excluded from the panel study if they did not participate in all parts. Thus, we excluded participants, if they canceled their participation in the panel study prior to onset of the first ES wave, did not take part in at least one of the two 14-day ES waves, received fewer than 10 beeps, for example, as a result of participation withdrawal during the ES waves, or did not react to the beeps (i.e., did not answer more than 5 beeps, or had an answer rate below 20%). These exclusion criteria were much less strict than the compensation criteria of the panel study (at least 14 beeps per ES wave); thus our study results do not apply only to "compliant" participants but can be generalized.

This resulted in a final sample of 592 participants, whose age ranged from 18 to 65 years with a mean of 41.7 years (*SD* = 12.9) with 55.3% of participants being male (*n* = 294) and 45.7% (*n* = 238) female. With respect to educational attainment, 0.6% did not have any degree, 15.4% had lower secondary education, 34.6% had junior secondary education, 29.3% did their A-levels, 19.5% graduated from university, and 0.6% had a PhD.

### Measures

To handle the vast amount of data assessed via traditional self-reports, mobile sensing, and ES, we applied a predictive modeling approach, using various machine learning (ML) algorithms. Following ML terminology, we refer to the outcome variable as the target variable and the predictor variables as features.

#### Target: missed beeps

The response to ES questionnaires (short: beeps) served as the target variable. To avoid artificially provoking smartphone usage, participants only received beeps upon actual usage of their smartphone. A beep was considered as *answered* and therefore coded as 1 if the participant opened the ES questionnaire in the app within 15 minutes after the respective notification and completed the ES questionnaire within further 15 minutes after opening the app. Whenever a participant received a beep but failed to answer it, a beep was considered as *missed* and coded as 0. These included instances in which participants intentionally chose not to respond to a beep, such as by wiping away the notification, or in which participants did not respond to or finish an ES questionnaire within the 15-minute time limit. If however, a participant did not use the smartphone after a scheduled beep within the associated randomly selected time window and therefore did not receive a notification for the ES questionnaire, this case was not considered in our analysis.

#### Features: person, context, and behavior

We extracted a total of *402* features. Not to exceed the scope of this report, we describe categories of extracted features with selected examples. However, a complete list of all features including a short description and the code that was used for feature extraction can be found as supplementary material in our OSF repository.

**Fig. 1:**
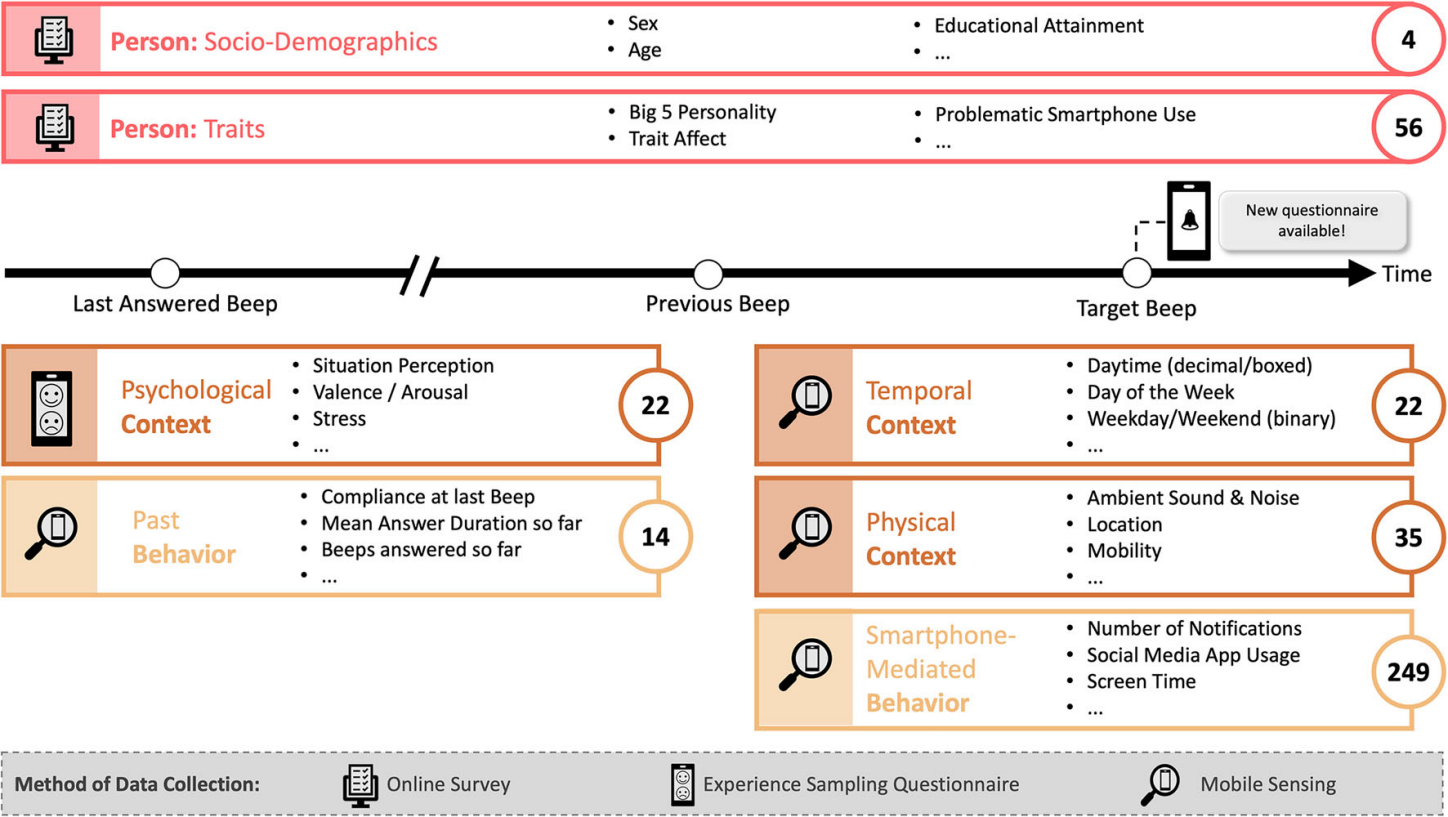
Overview of Features According to Categories and Data Collection Methods. *Note*. We were interested in compliance at the observational level, that is, how participants responded to a single beep, the target beep, at a given time. To this end, we used information about a participant’s response to preceding beeps and information on the current beeps, as represented by the timeline. We assigned this information to different (sub)categories, represented by the colored boxes with selected feature examples. The icons on the left of the boxes depict the respective data collection method. Numbers on the right of the boxes indicate the total number of features for each subcategory. In all, 402 different features were extracted for each beep

**Table 1:** Overview of self-report measures of the feature category person

Measure	Instrument	Included Features	Reference
Socio-demographics	Self-created	4 single items	
Big five personality	BFSI	5 factors and 30 facets	Arendasy et al. (2011)
Media usage habits	Self-created	12 single items	
Technology affinity	TA-EG	4 facets	Karrer et al. (2009)
Problematic smartphone usage	Self-created^a^	3 single items	Lee et al. (2014)
Trait affect	PANAS^b^	2 facets	Watson et al. (1988)

*Note.* List of questionnaires used for the assessment of socio-demographics, traits, habits, and preferences. A complete list of single items can be found in the study protocol of the SSPS (Schoedel & Oldemeier, 2020)

^a^ Items were selected and translated from Lee et al. (2014)

^b^ German translation by Breyer & Bluemke (2016)

We assigned our features to three main categories: *Person, Context*, and *Behavior* (see Fig. 1). Person features were assessed via the monthly online surveys. Some of the context features were assessed via ES questionnaires. The major part of the context as well as the behavior features were extracted from mobile sensing data assessed via the PhoneStudy app. Raw sensing data were logged as time-stamped data points stored with data type-specific information (e.g., app name for app usage logs, decibel values for ambient sound logs, longitude and latitude for GPS logs). User-smartphone interactions such as phone or specific app usage, notifications, or screen status were logged *event-based*, i.e., the app recorded data points whenever they occurred. In comparison, GPS data were logged *interval-based*, that is at fixed time points every 10 to 60 minutes, depending on the respective smartphone model. Ambient sensor data such as sound or light were also logged on an interval basis but only between 6 p.m. and noon so as not to put too much strain on the battery. To obtain an accurate picture of the user’s physical context while conserving battery power, GPS and physical activities were additionally logged *change-based*, that is, whenever a change was detected. To do so, GPS and physical activity features were gathered via the Google Fence API^5^, the Google Snapshot API^6^, and the Google Activity Recognition API^7^. For more details on logging procedures see also Schoedel et al. (2023).

##### Person

The category *Person* comprises features related to participants’ self-reported socio-demographics, traits, habits, and preferences. More specifically, we included socio-demographics, personality traits, media usage habits, technology affinity, problematic smartphone usage, and trait affect (see Table  1 for an overview of all measures). As problematic smartphone usage was assessed repeatedly in each of our six monthly online surveys, we used those assessments closest to the respective ES wave (i.e., online surveys assessed in the third and fifth study month for the first and second ES wave, respectively).

##### Context

The category *Context* comprises features related to the participant’s situation when receiving a beep. The three subcategories *Temporal Context*, *Psychological Context*, and *Physical Context* focus on different levels of abstraction.

##### Temporal context

Features included in this category characterize the time at which participants received beeps. We extracted features from the timestamps automatically recorded when the beeps were sent via the PhoneStudy app. This included the encoding of information on time as both decimal number and daytime category. For example, the timestamp 10:30:00 a.m. (Central European Summer Time or UTC + 2) was encoded as numerical (i.e., as 10.5) and categorical (in 2 hour time-boxes). In addition, time was encoded as information on day (weekday/weekend).

##### Psychological context

Features of this category describe the psychological context in which a beep was sent. As we do not have self-reported psychological context features if a beep was missed, we used the participant’s self-report at the last answered beep as a proxy. Measures included in this category were (1) a two-item state affect rating according to the Circumplex Model of Mood (i.e., valence and arousal; Russell , 1980) and (2) a single-item stress rating. Participants were asked to rate all three items on a 6-point Likert scale. In addition, we included (3) a single-item rating for each of the eight situational DIAMONDS (Duty, Intellect, Adversity, Mating, pOsitivity, Negativity, Deception, and Sociality) asking participants how they perceived the current situation (Rauthmann et al., 2014). Participants rated on a binary scale for each dimension of situation perception if it applied or not (Rauthmann & Sherman, 2018).

##### Physical context

Features from this category describe the current physical context, including location, mobility, and environmental cues at a very fine granular level (e.g., ambient light or sound). As these features are enabled by the mobile sensing component of the PhoneStudy app and therefore do not require active logging by participants, they were assessed regardless of whether participants responded to a beep. These features were derived using GPS, activity, light, and sound sensor data. These raw data were preprocessed according to a set of preprocessing pipelines. For example, GPS points were clustered per participant in order to identify each person’s home and workplace coordinates (i.e., the center of the cluster in which a participant was present most frequently between 1 a.m. to 5 a.m. for home vs. 10 a.m. to 4 p.m. on weekdays for workplace). Subsequently, we classified whether a person was at home or at work for a given beep based on actual GPS coordinates and their accuracy logged right before the respective beep. Additionally, we calculated, for example, the distance from home, whether the participant was likely to be traveling (e.g., by car or train), or whether they were in a bright or noisy environment at the time a beep was sent. To get a comprehensive picture of the physical context at the time a beep was answered or missed, we chose a time window of 60 minutes before the respective target beep to aggregate the raw sensing data points for this feature category.

##### Behavior

Features included in the category *Behavior* describe active behaviors in the time window before receiving a beep.

##### Smartphone usage

This category includes features on participants’ smartphone-mediated behaviors. Features were extracted based on the timestamped smartphone logs within 60 minutes before the respective target beep. We included general smartphone usage (e.g., time spent on screen, number of incoming calls), smartphone notifications (e.g., number of notifications, latency between receiving notifications and unlocking the smartphone), and app usage (e.g., Communication, Photo, News, or Music). Single apps were categorized to psychologically meaningful categories based on the system proposed by Schoedel et al. (2022). Thereby, we followed the proposed inclusion of app categories with sufficient inter-rater agreement (i.e., Cohen’s kappa > .60).

##### Past behaviors

This feature category is associated with participant responses to past beeps. Features included in this category were, for example, the number of sent beeps up to the time of the respective target beep or the mean answer latency, that is, the average time between receiving a beep notification and the time when the participant started answering the respective ES questionnaire. It also comprised the mean answer duration (i.e., the average time needed for completing ES questionnaires), the mean answer rate, and whether the previous beep was answered. In these features we only coded information on behaviors that occurred *before* the respective target beep. We did this to design our prediction model (see next section for more details) to be applicable in real time in future ESM studies. That is, if we would like to apply our prediction model in a new study to predict whether a participant will respond to the next target beep, we would only have information collected up to that specific time point. In this case, features such as the overall answer rate in the study would not be available if a participant is only halfway through.

### Data analysis

#### Machine learning

We used the previously described features (in total, 402 before and 190 after target-independent preprocessing) to predict whether a specific target beep was answered (or missed) at the observational level. This setting corresponds to a binary classification task. Machine learning predictions were conducted using the mlr3 environment in R (Lang & Schratz, 2023).

##### Preprocessing

We applied both target-independent and target-dependent preprocessing. The first included the replacement of extreme outliers in each feature (±4 standard deviations from the mean) by missing values. We applied this procedure to exclude anomalies in the data most likely caused by technical logging errors, while extreme expressions of features were preserved in the data. Further steps were the removal of features with more than 90% of values missing across all observations and the removal of features with zero or near-zero variance as defined by the default settings of the caret package (i.e., classification of a predictor as having variance near-zero if the percentage of unique values in the samples was less than 10% or if the ratio of frequency of the most common value to the frequency of the second-most common value was greater than the ratio of 95%/5%, Kuhn , 2008). All subsequent target-dependent preprocessing steps, namely scaling and missing data imputation, were integrated into the resampling procedure to avoid overfitting and leakage problems (i.e., information from the test set "leaking" into the training set in the prediction task). As some sensing components were only logged at specific time intervals throughout the day (e.g., ambient sensor data), some features showed a substantive amount of missing values. Imputation was conducted via histogram and tree-based learners, respectively, using the methods implemented in the mlr3pipelines package (Binder et al., 2021).

Regarding preprocessing, we also tested approaches to account for the class imbalance in our target variable such as the assignment of class-dependent weights or oversampling (Sterner et al., 2023) and model-specific hyperparameter tuning (e.g., lambda or mtry for elastic net and random forest, respectively). We reran the models without the described exclusion of extreme outliers (±4 SD of the mean) for which results are provided in the online materials in the OSF repository. However, none of these approaches led to considerable performance improvements but did considerably increase computational costs. Therefore, we report all results based on the default settings in the respected software packages for all hyperparameters.

##### Models

We benchmarked three models for the prediction task, namely (1) standard logistic regression, (2) elastic net regularized logistic regression (hereafter referred to as elastic net; Zou & Hastie , 2005) as implemented in the glmnet package (i.e., cv.glmnet; Friedman et al. , 2010), and (3) random forest (Breiman, 2001) as implemented in the ranger package (Wright & Ziegler, 2017). We selected these models as they facilitate the comparison of a familiar approach for classification in the behavioral sciences - ordinary logistic regression - with two more sophisticated, common machine learning algorithms, representing a regularized linear model (i.e., the elastic net) and a non-linear tree-based model (i.e., the random forest). Random forests consisting of many single decision trees automatically take into account interaction effects between variables, because the partitioning within a tree may depend on different predictor variables (for a more detailed introduction to random forest models, see Module 2 in Pargent et al. 2023). Elastic net models, on the other hand, are able to consider interaction effects only if explicitly stated in the model equation. However, we decided against including interaction terms in our analysis, as this would have enormously increased the (already large) number of predictors. Both algorithms are especially well-suited to the modeling problem at hand as they can handle identification or computation issues due to a large number of features and linear dependency among these (e.g., between Big Five factors and facets) (Dormann et al., 2013; Pargent et al., 2023; Hastie et al., 2009). Apart from the described models, we trained a baseline model that served as a reference point to benchmark the other models. This baseline model predicted the most common class of the target variable (i.e., that a given beep was answered) among all observations in the respective training set by assigning probabilities corresponding to the relative frequency of the class labels in the training set without considering any of the features (Lang et al., 2019).

##### Performance evaluation

We estimated the prediction performance for the different models by using 10-fold cross-validation with 10 repetitions (10x10 CV) as resampling procedure. Because the basic idea behind the ESM is to collect repeated measurements within individuals, we considered the assumption of independence of residuals to be violated. To account for the nested structure of our data, we applied blocked resampling with participants’ unique identifiers as the blocking variable. By using this blocked resampling strategy, we ensured that all observations of one individual completely went into either the test or the training data set but were never split up in order to counteract overoptimistic performance estimates (Dragicevic & Casalicchio, 2020).

**Fig. 2:**
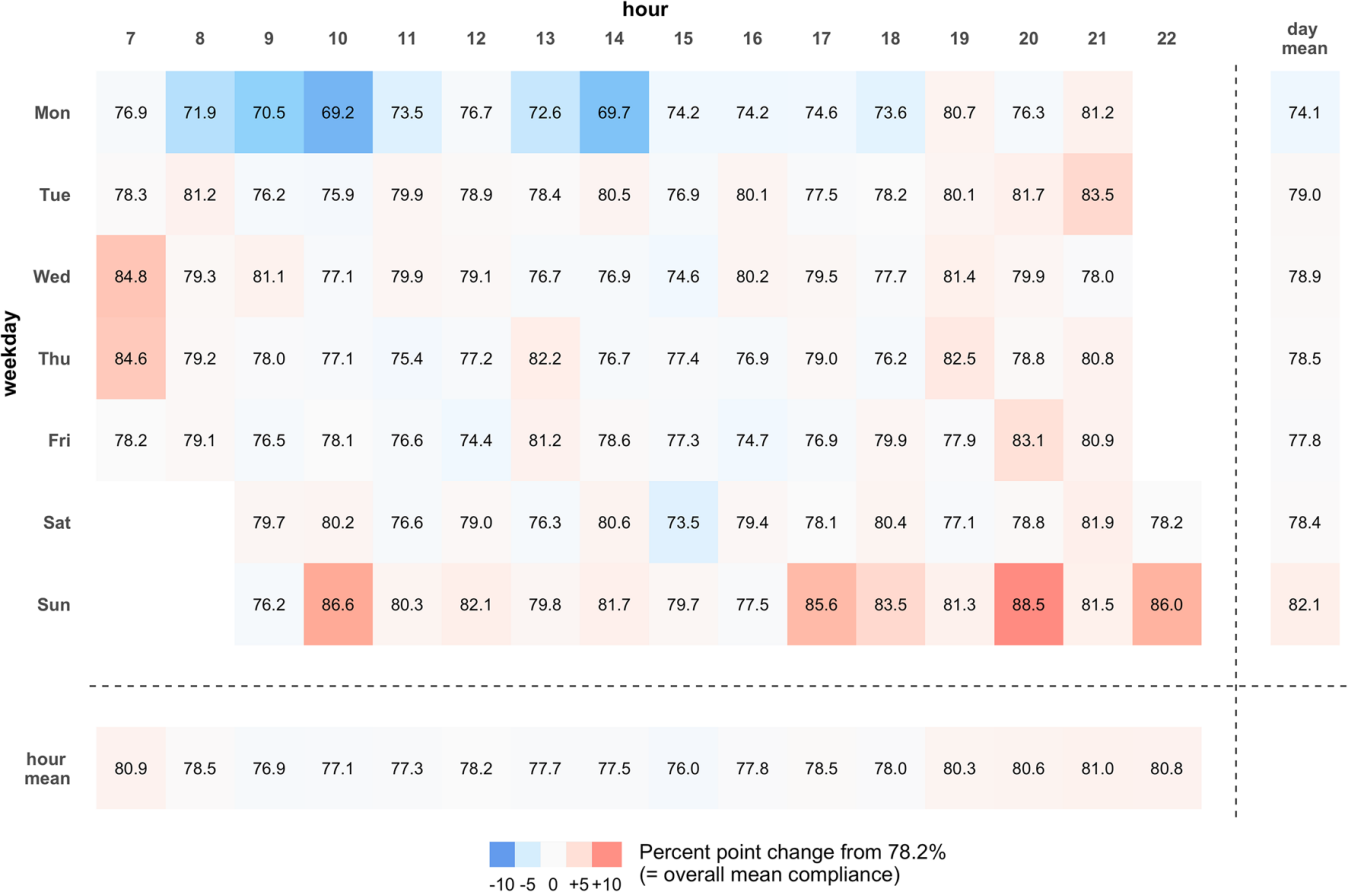
Deviation of beep level Compliance Rate from Overall Compliance Rate Depending on Weekday and Daytime. *Note.* Numbers in the grid cells represent the average compliance rate at the beep level across all participants for the respective weekday and daytime combinations. Daytimes of beeps ranged from 7 a.m./9 a.m. to 9 p.m./10 p.m. on weekdays and weekends, respectively. Right and bottom margin cells represent the beep level compliance rates averaged for the respective weekday and daytime. The degree of coloration represents the degree of deviation from the average overall compliance rate of 78.2%, with reds representing higher and blues representing lower compliance rates at the beep level

To evaluate our models’ performances, we used the area under the receiver operating characteristic (ROC) curve. In binary classification tasks, the ROC curve considers both, the true positive rate (sensitivity) and the false positive rate (1 - specificity) of a model to evaluate a model’s predictive ability as a function of different discrimination thresholds. Integrating over the ROC curve yields the area under the curve (AUC) metric, which can be thought of as an "integrated measure" between both sensitivity and specificity. The AUC can be interpreted as the probability of the model ranking two randomly selected beeps (one of each class, one answered, one missed) correctly (i.e., the calculated probability of being answered is higher for the answered beep than for to the missed beep) (Viaene & Dedene, 2005). Describing a probability, AUC values can range from 0 to 1. The AUC metric can be considered robust to class imbalance (Boughorbel et al., 2017). A naïve guessing approach, as applied by our baseline model yields an AUC of .50 (Fawcett, 2006). Accordingly, AUC values smaller and larger than .50 represent worse or better prediction performances than our baseline model, respectively. As a more intuitive performance measure for classification, we additionally report Matthew’s correlation coefficient (MCC), which is a method of calculating the Pearson product-moment correlation coefficient between actual and predicted values based on the confusion matrix (Chicco & Jurman, 2020). The MCC ranges from -1 to +1, equals zero for the baseline model’s predictions, and produces high scores only if good prediction results are obtained in all of the four confusion matrix categories (Chicco et al., 2021). This is why it can be considered a more reliable statistical measure compared to more popular metrics such as accuracy, especially for cases with strong class imbalance (Chicco & Jurman, 2020).

#### Model interpretation

To gain insights into the prediction models, we performed follow-up analyses by applying two interpretable machine learning tools. As a preview of our results, the elastic net model achieved the highest average prediction performance. Therefore, we decided to focus our interpretable machine learning analyses on this model and to use model-specific techniques exclusively for the elastic net model.

##### Single feature importance

We investigated which features were most predictive (i.e., informative) of answered beeps. Accordingly, we estimated standardized beta coefficients and used them as a metric for single feature importance. Due to the large number of features, we did this exclusively for the features that were selected by the elastic net model. In the elastic net’s feature selection, the regression coefficients of uninformative features are shrunken to zero. This is done based on shrinkage parameters that are selected using a model-inherent cross-validation. To account for the random component introduced by this cross-validation, we trained 100 separate elastic net models and calculated the rate of inclusion into the final models, the average beta coefficients, and the 10-90 percentile ranges for the average beta coefficients across these 100 iterations.

**Fig. 3:**
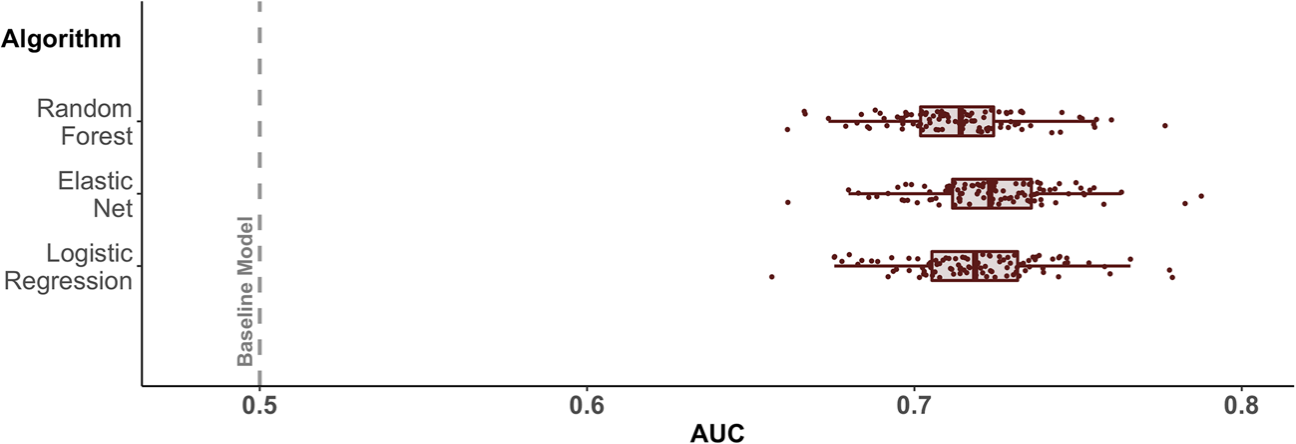
Prediction Performances Across Iterations of Repeated Cross-Validation. *Note.* Distribution of the area under the operating characteristic curve (AUC) across the resampling iterations of the applied 10x10 CV scheme for random forest, elastic net, and logistic regression models. The gray dotted line at an AUC of .500 represents the prediction performance of the baseline model. AUCs of the single iterations are represented by single dots. The boxes contain all values between the 25% and 75% quantiles. Their middle line indicates the median. For presentation clarity, the AUC scale was cutoff at .500 and .800

##### Grouped feature importance

As our features can roughly be clustered into the three categories (person, context, and behavior) with several subcategories, we were also interested in whether one of these subcategories was particularly relevant (i.e., informative) for our model’s predictions. Accordingly, we conducted a *leave-one-group-out* analysis by comparing the prediction performance of the elastic net model for the full feature set containing all features of all categories with its performance after the features of each of the different subcategories had been excluded. Again, we used a 10x10 CV scheme. If the AUC decreased after excluding a specific feature subcategory, this indicated that the respective feature subcategory was important for the prediction performance.

To anticipate our results, the *past behavior* and *physical context* features led to noteworthy decreases in prediction performance in the *leave-one-group-out* analysis. To further explore the importance of these feature subcategories, we additionally implemented a *leave-one-group-in* analysis. Thus, we trained two models that only used *past behavior* or the combination of *past behavior* and *physical context* feature for prediction.

As the *past behavior* subcategory was by far the most important one, we additionally considered the possible masking effect of this subcategory in relation to all other subcategories by implementing a hierarchical leave-one-group-out approach. Thus, we first excluded features of the subcategory *past behavior* from the feature set and then compared the prediction performance changes after excluding each of the other remaining feature subcategories. For example, one could assume that relevant associations between the personality trait conscientiousness could be related to compliance at a given beep but would then necessarily also be related to compliance at the previous beep. Accordingly, including compliance at the previous beep as a predictor would at least to some extent include, control for, or *mask* effects of the person trait. When, however, excluding the auto-regressive effect of compliance at the previous beep, effects of the personality trait conscientiousness could be detected.

## Results

### Descriptive statistics

Overall, participants received 26,750 beeps of which 20,907 were answered, aggregating to an overall compliance rate of 78.2%. At the level of single persons, participants received, on average, 45.2 beeps of which, on average, 35.3 were answered. With a value of 78.6%, participants who reported being male had similar average overall compliance rates as participants who reported being female, at 78.8%. Similarly, average overall compliance rates were comparable for different age groups: 77.8%, 79.0%, and 78.9% for participants aged 18-29, 30-49, and 50+ years, respectively.

In addition, as presented in Fig. 2, we descriptively investigated the average compliance rates at the beep level and their deviation from the average compliance rate at the overall level (i.e., 78.2%) separately for each combination of weekday and daytime in hourly time bins (see main figure area), for weekdays irrespective of daytime (see right margin), and for daytime irrespective of weekdays (see bottom margin).

On average, the compliance rate at the beep level was, in comparison to the compliance rate at the overall level, lower on Monday mornings (blue cells on the top left) and higher on mornings in the middle of the week and Sunday evenings (red cells in the middle left and on the bottom right). There were no noticeable deviations neither for specific weekdays, irrespective of daytime, nor for specific daytimes, irrespective of weekdays. Please note that due to our ESM design, these descriptive patterns have to be interpreted conditionally, that is, under the condition that participants had to actively use their smartphone in order to be notified of an ES questionnaire and, consequently, to be able to answer (or miss) a beep.

Not to go beyond the scope of this article, further descriptive statistics of our 402 features and their correlation with compliance at the beep level can be found in our OSF repository. Additionally, in the OSF online repository, we provide a descriptive overview of the total number of sent beeps across all participants for each day, daytime, and day $\times$ daytime combination.

**Table 2:** Top 20 important features in the elastic net models

			Standardized Beta Coefficients		
Variable	Group^a^	% Inclusion	*M*	*SD*	10-90% Percentiles
Mean Answer Rate (so far)	B2	100	0.50	0.01	[0.49; 0.51]
Mean Answer Latency (so far)	B2	100	-0.14	0.01	[-0.14; -0.13]
Compliance at Last Beep (binary)	B2	100	0.12	0.01	[0.11; 0.13]
Participant at Home (GPS)	C3	100	0.10	0.01	[0.09; 0.11]
Number of Missed Beeps Prior	B2	100	-0.08	0.01	[-0.09; -0.07]
Participant in Rail Vehicle	C3	100	-0.07	0.00	[-0.08; -0.07]
Age	P1	100	0.04	0.01	[0.03; 0.05]
Participant at Work (GPS)	C3	100	0.04	0.01	[0.02; 0.05]
Number of Events Louder Than 55 db	C3	100	-0.04	0.01	[-0.05; -0.03]
Number of Unique Apps Used	B1	100	0.03	0.01	[0.02; 0.04]
Tech.-Enthusiasm (Tech.-Affinity Subfacet)	P2	100	-0.03	0.01	[-0.04; -0.02]
Number of Unique App Categories Used	B1	100	0.03	0.01	[0.02; 0.05]
Duration of Finance Apps Used	B1	100	0.03	0.01	[0.02; 0.04]
Weekday (1=Monday)	C1	100	0.03	0.01	[0.02; 0.03]
Answer Latency at Last Answered Beep	B2	100	-0.02	0.01	[-0.04; -0.01]
Min. Latency of App Notification Usage	B1	96	0.02	0.01	[0.01; 0.04]
Number of App Usages	B1	99	0.02	0.01	[0.01; 0.03]
Dutifulness (Conscientiousness Subfacet)	P2	96	0.02	0.01	[0.01; 0.03]
Number of Events Brighter Than 10 Lumen	C3	99	0.02	0.01	[0.01; 0.03]
Participant in 4-Wheel Vehicle	C3	100	-0.02	0.00	[-0.02; -0.02]

*Note.* Table of top 20 features as identified from 100 iterations of elastic net model. Features are ordered with respect to their mean standardized beta coefficient across all iterations in which they were included into the model. Column % Inclusion indicates in how many iterations the latter was the case (i.e., coefficient was not shrunken to 0). Criterion for inclusion of features in this table was an inclusion rate of at least 95% (i.e., feature was selected in at least 95 elastic net iterations) or an absolute mean standardized beta coefficient equal to or greater 0.03

^a^Group column indicates feature category:

P1 = Person: Socio-Demographics, P2 = Person: Traits,

C1 = Temporal Context, C2 = Psychological Context, C3 = Physical Context,

B1 = Smartphone Usage, B2 = Past Behavior

### Prediction of compliance at the beep level

Our central research question was whether there are characteristics that are systematically associated with compliance at the beep level. We applied a machine learning approach to condense the information in our large set of investigated features. To briefly summarize, we did find indications for a compliance bias at the beep level. We compared different models, and they all performed better than our baseline model (AUC = .500). That is, the models were all able to grasp systematic variance in the collection of *person*, *context*, and *behavior* features to make predictions for whether participants answered a beep. In comparison, the standard logistic regression model ($M_{AUC}$ = .719), the elastic net model ($M_{AUC}$ = .723), and the random forest model ($M_{AUC}$ = .713) achieved similar mean prediction performances, but the elastic net model slightly outperformed the other two. The distributions of prediction results across the 100 resampling iterations resulting from our applied 10x10 CV scheme are depicted for all models in Fig. 3.

When using the MCC as the performance evaluation metric, the linear models ($M_{MCC}$ = .217 for standard logistic regression and $M_{MCC}$ = .194 for elastic net regression) also outperformed the non-linear random forest model ($M_{MCC}$ = .129). Moreover, all three models were better than the baseline model ($M_{MCC}$ = .000).

### Interpretation of compliance predictions at the beep level

Having found indications for a compliance bias at the beep level, we conducted a follow-up analysis to explore which characteristics in particular were predictive of whether participants missed a beep. As mentioned, we only considered the elastic net model, which had the highest AUC in the benchmark.

**Fig. 4:**
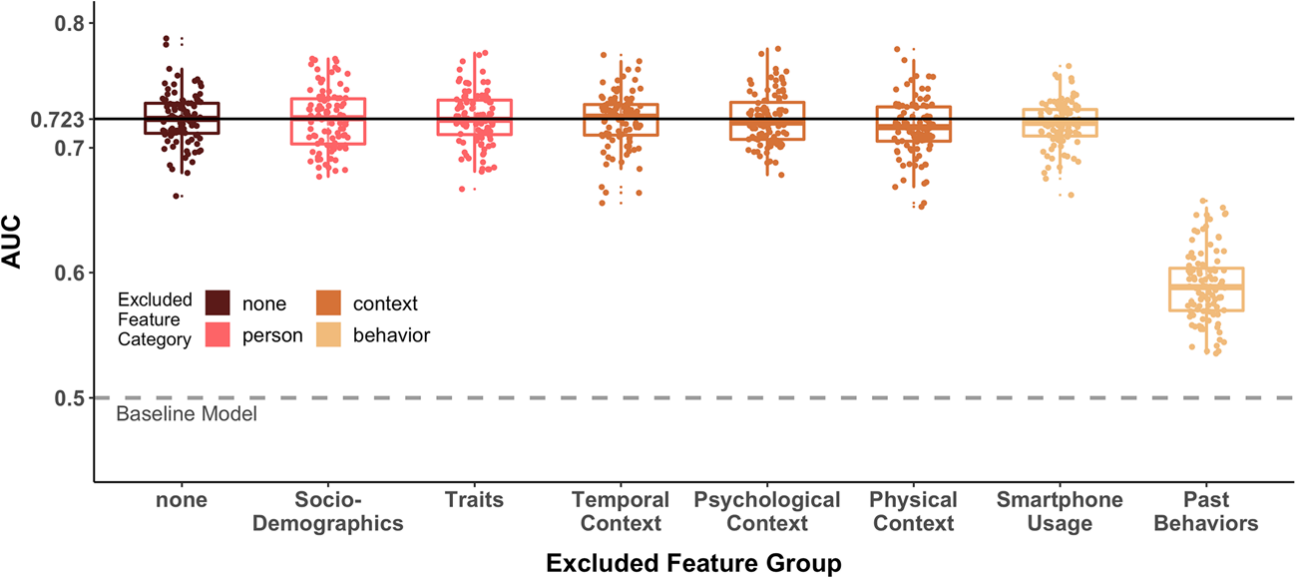
Prediction Performance of Elastic Net Models After Exclusion of Specific Feature Groups. *Note.* Distribution of the area under the operating characteristic curve (AUC) across the resampling iterations of the applied 10x10 CV scheme for the elastic net models after exclusion of each single subcategory. The boxplot in dark brown represents prediction performances with the initial full set of features (i.e., no exclusion of categories). For clarity, we include the median performance of this model as a solid black line. The remaining boxplots represent the performance (ordered and colored by feature subcategories) when one of the subcategories was excluded in each case. The gray dotted line at an AUC of .500 represents the prediction performance of the baseline model

#### Features in their individual role

We trained 100 elastic net models and, for each model, examined which features were most important in terms of their absolute mean standardized beta coefficients. Based on this, we extracted the top 20 features across the 100 models. In doing so, we found that many features (more than 200) were equally important, that is, they had the same mean absolute beta values. Therefore, we applied more strict selection criteria and only included features that had an average standardized beta coefficient > 0.05 or were included in at least 90% of the 100 elastic net models. We present the resulting list of the top 20 features in Table 2.

The table shows that features of all categories and subcategories (except *psychological context*) were represented among the 20 most important features. Features of the category *past behavior* particularly stood out, as the *mean answer rate (so far)* had the highest averaged standardized beta coefficient and was by far the most informative feature for the elastic net model’s predictions. The two next most important features (*mean answer latency (so far)* and *compliance at last beep*) were also from the category *past behavior*.

Features of the category *physical context* were also represented frequently among the top 20. Accordingly, features such as whether participants were at home or at work, in an environment louder or brighter than a specific decibel or lumen value, or in a rail or 4-wheel vehicle were consistently included in the elastic net models, with mean standardized beta coefficients ranging up to 0.10. Apart from these, weekday was the only feature of the *temporal context* feature category appearing in the top 20.

Comparing the higher level categories of *behavior*, *context*, and *person*, the latter was the least represented among the top 20 features. *Socio-demographics* and *traits* were, on average, among the features with the lowest standardized beta coefficients and inclusion rates. All person features included in the top 20 (i.e., age, dutifulness, and technology enthusiasm) had standardized beta coefficients below .05.

#### The role of features as groups

Besides considering individual features, we also explored their informativeness in their group constellations. Note that we assigned our features to seven subcategories of *person*, *behavior*, and *context* characteristics. Figure 4 shows the relevance of each subcategory by plotting the average prediction performance (quantified by the AUC across the resampling iterations of the applied 10x10 CV scheme) when each feature category was excluded. We found the largest decrease in prediction performance with the exclusion of the category *past behavior*. The average prediction performance decreased from $M_{AUC}$ = .723 (when the category was included for prediction) by .134 to $M_{AUC}$ = .590 (when the category was excluded for prediction). The exclusion of other feature categories resulted in smaller changes in the prediction performance, with decreases of .007 for the category *physical context*, followed by .004 for the category *smartphone usage*. Excluding features of the categories person (*socio-demographics* and *traits*), *temporal context*, or *psychological context* resulted in average decreases of below .001.

In summary, features from the category *past behavior* were by far most important in predicting compliance at the beep level, followed by features of the category *physical context*. Thus, the results of this leave-one-group-out analysis are in line with both the single feature importance analysis and the leave-one-group-in analysis. In the latter approach, we trained two additional models using *only* the features of the category *past behavior* or a combination of the categories *past behavior* and *physical context*. Both models produced comparable results to the model with features from all categories, with average prediction performance decreasing slightly by .013 and .005, respectively, compared to the full model.

To obtain further insight into which features are relevant for predicting compliance in the ESM setting, we also explored a possible "masking of effects" by the category *past behavior*. That is, we were interested in whether other feature categories are important beyond the dominant category of *past behavior*. To this end, we reran the leave-one-group-out analysis. This time, however, the full model included all features except the *past behavior* category, and in each leave-one-group-out model, one of the other subcategories was additionally excluded. We found that the resulting models achieved lower overall prediction performances compared to the original prediction models, with $M_{AUC}$ ranging from .577 to .590. However, there were no major changes in the rank order of the different feature subcategories. Features of the categories *physical context* and *smartphone usage* were still the two most important feature subcategories and *traits* remained to carry very low predictive information. We present the results for this additional analysis in the supplemental material in our OSF repository.

## Discussion

The present study was designed to provide new insights into characteristics related to compliance and consequently the nature of missing data in ESM studies. To this end, we adopted a multi-method approach and combined various *person*, *behavior*, and *context* features collected via surveys, ES questionnaires, and passive mobile sensing to predict compliance at the beep level in an ESM study with 592 participants and more than 25,000 observations collected over several weeks. We used machine learning techniques and found empirical indicators of a compliance bias. Using the interpretable machine learning toolbox, we explored which characteristics were most informative in predicting compliance at the beep level individually and as aggregated feature categories. Features from the subcategory *past behavior* were by far the most relevant, followed by features in the category *physical context*. *Person* and *psychological context* features were of least importance. In the following sections, we discuss our results and how they help shed new light on compliance in ESM studies.

### Predictability of compliance in ESM studies at the beep level

We found that participants’ actual behavior - namely whether a beep was responded to or not - was predicted above chance in our ESM study. That is, we found systematic associations between compliance at the beep level and *person*, *behavior*, and *context* features. Each of the three ML models outperformed the baseline model. The linear models (standard logistic regression and elastic net) were not inferior to the non-linear random forest model which has already been observed in previous literature applying machine learning approaches to psychological research questions (Schoedel et al., 2023; Christodoulou et al., 2019; Pargent et al., 2019). One possible explanation for this could be that the true underlying associations are indeed linear and as such could be captured somewhat better by the linear models than by the non-linear random forest model, which can only approximate smooth or linear relationships (Hastie et al., 2009; Grömping, 2009). In addition, non-linear models have problems capturing truly non-linear relationships when measurement error is present in the predictor or outcome variables (Jacobucci & Grimm, 2020). Because some of our features were psychological constructs, this reasoning may also have applied to our study and thus also limited the potential of the non-linear random forest model. Simulation studies showed that this effect is even exacerbated with smaller samples as the linear model is more impervious to sample size (Jacobucci & Grimm, 2020). Our study with 592 participants and more than 25,000 beep level observations would have been among the largest 3% according to a recently published meta-analysis examining compliance in ESM studies (Wrzus & Neubauer, 2022). However, in the machine learning context, sample sizes of several thousand people are not uncommon (Rosenbusch et al., 2021).

To compare the ranking of our models’ performance relative to previous studies, we first considered commonly used rules of thumb. With mean performance metrics (AUC) exceeding .700, our models performed at a level which would be considered acceptable (Hosmer et al., 2013). We additionally inspected the strength of the association between the actual and the predicted response to beeps as a further evaluation metric. The correlations ranged between .129 and .217, so they were low to medium. To summarize, our models were able to predict compliance at the beep level, but the prediction performance was far from perfect. Thus, despite using a large variety of *person*, *behavioral*, and *context* variables, we found little compliance bias at the beep level. However, given the context - an increasing number of ESM studies across disciplines - even a small compliance bias could be meaningful for the validity of research findings (Götz et al., 2022). Accordingly, the magnitude of research findings biased due to missing data could be considerably decreased if researchers across disciplines explicitly considered compliance bias in ESM studies, for example by including control or auxiliary variables to statistically counteract (Newman, 2014).

Second, we also wanted to compare our results more specifically with effects found in psychological studies addressing similar research questions. This proved to be a challenging task, however, as most previous studies have used an explanatory modeling framework rather than a predictive one (Sun et al., 2020; McLean et al., 2017). They reported in-sample effects, but we evaluated our models out-of-sample, or how they performed on resampled, and thus unseen, observations when predicting compliance at the beep level (Shmueli, 2010). While explanatory modeling is an important strategy to gain a better understanding of psychological processes, psychology as a research discipline has been criticized as strongly focusing on explanation but neglecting prediction (Yarkoni & Westfall, 2017). By combining ideas from explanatory and predictive modeling, psychology has the opportunity to extend its focus and thus increase the generalizability and reproducibility of research results (Hofman et al., 2021). Our study contributes to this debate (Yarkoni & Westfall, 2017; Rocca & Yarkoni, 2021) by applying predictive modeling and aiming at the accurate prediction of actual response behavior. This data-driven approach can help in developing ideas for underlying (causal) mechanisms or generating new hypotheses for explanatory modeling (Shmueli, 2010). Especially for the objective of the present study – the identification of variables linked to participants’ missing beeps – predictive modeling was a useful approach because it allowed us to condense information included in a broad set of multi-methodologically collected variables.

### Differential importance of person, behavior, and context features

Because our models were able to systematically grasp variance in the large set of *person*, *behavior*, and *context* features, we explored which features were related to whether participants missed specific beeps.

#### Past behavior predicts future behavior

The results of our follow-up analyses provided consistent evidence that study-related past behaviors were most relevant for predicting compliance at the beep level: In particular, participants’ average preceding compliance was by far the most informative feature. Considered individually, the top three most relevant features for predicting compliance belonged to the feature category *past behavior*. In the leave-one-group-out analysis, the category *past behavior* was also by far most important. To illustrate, the decrease in prediction performance related to the exclusion of all features of this category was higher than the sum of performance decreases caused by excluding all other feature categories individually. Moreover, the leave-one-group in analysis showed that a model only considering *past behavior* features was able to achieve a prediction performance only slightly inferior to the full model including information of all features from all categories.

The importance of (study-related) past behavior for predicting compliance behavior at the beep level is in line with a "classic" finding in psychology: Past behavior predicts future behavior (Ouellette & Wood, 1998; Albarracin & Wyer, 2000). This has been found consistently in different areas, such as blood donation, physical exercise, or voting to name but a few (Rogers & Aida, 2011; Rodrigues et al., 2021; Ferguson & Bibby, 2002). According to previous literature, "well-practiced behaviors in constant contexts recur because the processing that initiates and controls their performance becomes automatic. Frequency of past behavior then reflects habit strength and has a direct effect on future performance" (Ouellette & Wood , 1998, p. 54). Applied to the ESM setting of our study, this could mean that beep level compliance behavior might have become automated over time in the constant ESM study setting and thus proved to be the most informative predictor.

In contrast to past (study-related) behaviors, smartphone use such as calls or app use immediately before a certain beep was far less relevant for predicting compliance in ESM studies - both individually and when considered as a feature category. The number of unique apps used (in the past 60 minutes) was the most important feature from this category and could be considered a proxy for diversity of smartphone use. However, single feature effects from this category were very small. This could be related to the fact that there was some asymmetry in the resolution of the target behavior (i.e., snapshot at a specific time point) and the extracted features (i.e., snapshots aggregated over 60 minutes). However, it would also be plausible that digital smartphone use represents a different class of behavior than (analog) study-related behaviors and is therefore less informative for predicting compliance at the beep level.

#### Physical context matters (a bit)

*Context features*, particularly those related to *physical context*, played some role – albeit a much smaller one compared to *past behavior*. In line with this, the leave-one-group-in analysis showed that using a combination of *past behavior* and *physical context* features without consideration of all other feature groups achieved a prediction performance that can be considered equivalent to the full model containing information of all features. In more detail, information on whether a participant was at home at the time of receiving a beep was among the most informative features for predicting (non-)compliance. More precisely, being at home was associated with a higher probability of responding to a given beep. Similarly, being at work was associated with a higher probability of answering a beep. Both GPS-based location features have in common a relatively low mobility. That is, participants usually stay at home or at work for relatively long periods. Thus, our results might indicate that features associated with low mobility are associated with a higher probability of responding to a given beep. In line with this interpretation, features of high mobility such as being on a train or in an automobile were associated with a lower probability of responding to a given beep in our study. Overall, this finding is in line with previous studies that found increased compliance when participants stayed at specific locations (e.g., when being at food places or at home; Boukhechba et al. , 2018; Rintala et al. , 2020) and decreased compliance when participants had a higher level of physical activity (McLean et al., 2017).

Besides features informing about mobility, other contextual features were informative for predicting compliance, especially those enabling a high resolution of physical surrounding. For example, the number of events louder than 55 decibels or brighter than 10 lumens in the hour before a specific beep were among the top 20 predictors for compliance at the beep level. Thus, *physical context* was related to whether participants reacted to beeps. Note, however, that this finding has to be interpreted with caution, as ambient noise and sound were only measured between 6 p.m. and noon in our study and therefore might have been confounded with temporal information that was assigned to the category *temporal context*. For time features, we found patterns contrasting to previous studies (e.g., Rintala et al. , 2020; Csikszentmihalyi & Hunter , 2003). For example, Csikszentmihalyi and Hunter (2003) found decreased compliance rates on Sundays, whereas our study found compliance to increase with progression of the week from Monday to Sunday (indicated by inclusion of the feature *weekday* in the top 20 and its positive standardized beta coefficient). Likewise, on a descriptive basis, Mondays and Sundays were the days with the lowest and highest average compliance respectively in our study. This result, which contrasts with previous literature, may be related to our scheduling and notification approach. In our study, participants were only sent a beep if they actually used their smartphone in the time interval after a scheduled beep. We applied this procedure to capture natural smartphone behavior (van Berkel et al., 2019). Therefore, our participants received beeps only if they had time to use their smartphone, irrespective of day. On free days, such as Sundays, they might have had more time to respond to a beep than on work days. When participants received beeps on Monday mornings, they might have been more likely to dismiss it as they probably used their smartphones, for example, to work through their after-weekend e-mails at work thus experiencing a higher level of stress and therefore responding to fewer beeps (Pindek et al., 2021).

#### The minor role of psychological features

Finally, the included psychological features contributed little to predicting compliance at the beep level. In more detail, in the *person* category, only age, technology enthusiasm (a subfacet of technology affinity), and dutifulness (a subfacet of the Big Five dimension conscientiousness) were informative, albeit at comparatively low levels. *Psychological context* features such as mood or stress were not among the 20 most important features. Accordingly, removing the categories of *socio-demographics*, *traits*, and *psychological context* in our grouped feature importance analyses resulted in a negligible reduction in prediction performance. This was the case even when the *past behavior* features were removed first and then additionally the *trait* features, arguing against a masking effect of the past behavior features. Our results for person features are in line with previous research, which has also found no or at most very little systematic non-response bias introduced by person characteristics such as personality traits (Courvoisier et al., 2012; Sun et al., 2020).

Regarding *psychological context* features, our results are also in line with previous studies identifying null findings (e.g., Rintala et al. , 2020). However, it should be noted that previous results in this area are inconsistent: Some studies have also found small effects for some psychological context variables (e.g., feeling stressed, upset, or enthusiastic; Murray et al. , 2023; Silvia et al. , 2013). One reason for this ambiguity in previous research could be that the effects for psychological context features might be very small, if present at all, and additionally be methodologically masked. As psychological context features rely on self-reports, this information is missing for a point in time if participants do not respond to the beep. As a workaround, researchers usually use the psychological context information reported in one of the previous beeps to predict compliance (Silvia et al., 2013; Rintala et al., 2020; Sokolovsky et al., 2014). Thus, the included psychological contextual information frequently refers to the participant’s psychological state hours before. But as psychological states are highly fluctuating (Fleeson, 2001; Heller et al., 2007), this category of features might be little informative for compliance prediction.

### Study compliance as a trait?

In summary, a key finding of our study is that *past behavior* features are by far most important for predicting compliance at the beep level. If past compliance behavior predicts future study behavior, this, in turn, leads to the question of whether compliance in ESM studies might be some sort of temporally stable person-level trait. Based on our analyses we cannot rule out the possibility that an actually unobserved (psychological) trait drove our compliance prediction and past behavior is just a kind of observable manifestation of this trait. For example, a person with a high score on the (unobserved) compliance trait, might also be more likely to respond to both the last and the given beep. Thus, as this (unknown) trait was not explicitly considered as predictor, a direct relation with compliance could, of course, not be observed in our study. Nevertheless, this trait could have effects on compliance, as the *past behavior* features might have carried over its effect. Please note that this is only our post-hoc interpretation and future studies should investigate this assumption, for example, by theory-guided derivation of new constructs or inclusion of known constructs (e.g., specific motivational aspects) in future beep level prediction studies. One additional way to further investigate the assumption of a stable person-trait, would be the use of a measurment burst design. By collecting ESM data during multiple ESM periods (bursts) at different times, stable compliance rates within participants would give some further support to this idea. For the sake of simplicity, we have referred to one single compliance trait in this paragraph. But future research should also investigate if one or maybe even several traits underlie *past behavior* features.

Our study gives a starting point for the search of a compliance trait by limiting the range of eligible constructs. If there is a compliance trait, it seems to be mostly independent of "traditional" psychological traits such as personality or attitudes. Even after excluding the *past behavior* features in our study, we found no considerable decrease in the prediction performance when additionally dropping the *trait* subcategory. Thus, we conclude that the effect of the traits included in our study was not masked by the effect of the past behavior features. The compliance trait might therefore carry different content information or have a less abstract resolution than, for example, established personality traits such as conscientiousness. At the same time, the finding that the included psychological traits (and states) were not related to compliance at the beep level, is rather good news for research disciplines such as personality psychology. The subject of interest such as personality traits or affect states seem not to be strongly and systematically related to missing data in the ESM setting.

### Implications for applied and methodological research

Our findings come with implications for both researchers applying ESM in their empirical studies and methodological researchers investigating ESM as their subject of research.

For researchers applying ESM, our results could help to optimize participant compliance at the beep level. For example, if researchers want to know whether a participant took their medication on a particular day (Verhagen et al., 2016), a promising approach to monitor treatment in clinical trials might be to send beeps only in contexts in which participants are most likely to respond, such as when they are at home or at work but not when they are on a train or in a car. A limitation of this compliance optimization approach is, however, that the core idea of ESM (i.e., random sampling across situations, moods, and experiences in everyday life) gets lost. This strategy should therefore be treated with caution, as the randomness of the sampling is arguably restricted when using this compliance-optimized approach. By selecting only the contexts in which participants are most likely to respond to beeps, researchers are likely to introduce a new type of bias, as some specific contexts are already selectively excluded during data collection (Lathia et al., 2013; van Berkel & Kostakos, 2021). Thus, researchers should be aware of the trade-off between optimizing compliance rates on the one hand but also keeping the idea of random sampling in their ESM studies on the other.

Second, our study provides ESM researchers with a guide on which variables to consider as control or auxiliary variables. This could help bring them one step closer to the (desired) goal of missing data at random (MAR) and at the same time one step away from biased study results and errors due to non-compliance bias (Newman, 2014). Based on our results, potential candidates for such control variables are information on participant mobility at the time of receiving a beep (e.g., being at home or at work versus being in a rail vehicle). This information could be operationalized through passive GPS tracking. In this context, developments in smartphone technologies are increasingly facilitating the collection of mobility data for research purposes (Müller et al., 2020; Harari et al., 2016; Miller, 2012).

In addition, scholars have recently highlighted the enormous potential of using mobile sensing for investigating compliance in ESM studies (Murray et al., 2023; Sun et al., 2020). As far as we know, our study is one of the first to respond to this call and thus could also serve as a starting point for future methodological research focusing on ESM as a research subject.

First, one possible objective of future research could be to gain a more thorough understanding of the above-mentioned differences between compliance-optimized vs. randomness-optimized approaches applied in ESM studies. One and the same research question could be addressed by collecting data via both approaches and comparing findings, depending on the applied optimization scheme. Moreover, irrespective of the subject of research, effects on compliance could be investigated by experimentally manipulating the type of optimization approach. This comparison, in turn, might help in understanding the possible (intended or unintended) impact of researcher degrees of freedom on findings in ESM studies, such as biases due to study design aspects related to compliance, such as suspending ESM beeps on specific weekdays (Wicherts et al., 2016).

Second, future methodological research could extend our approach of combining ESM and mobile sensing in several important ways to see how robust compliance bias at the beep level in ESM is across different study settings. On the one hand, future studies could apply different ESM designs and investigate if compliance biases depend on the degree of invasiveness of the used ESM schedule (van Berkel et al., 2019). On the other hand, studies could include additional feature categories such as physiological parameters (e.g., heart rate or stress measurements from smartwatches or other wearables). A broader set of included features beyond person, behavior, and context characteristics could further contribute to understanding compliance in ESM studies (Wrzus & Mehl, 2015) and could further increase the prediction performance obtained in our study. Finally, future studies could also compare participants’ perception of compliance and their reported reasons for missing beeps with their actual compliance behavior and reasons deduced from objective data to gain further insight into compliance in ESM studies.

### Limitations

The present study encountered some limitations. First, our ESM scheme deviated from more "traditional" time-contingent designs reported in the literature. This deviation should be considered when interpreting our results. In most previous ESM studies, participants received a fixed number of notifications at fixed or (quasi-)random times prompting them to respond to a beep (Wrzus & Neubauer, 2022). In contrast, we used an ES scheme that could be considered a combination of time-contingent and event-contingent sampling (Reis et al., 2014): Beeps were scheduled pseudo-randomly, that is, they were time-contingent in pre-specified intervals across the day but only triggered if participants turned their screen on within a particular time interval. Thus, participants were not proactively notified, for example, via the smartphone’s vibration or acoustic signals. Instead, they only received a beep when they used their smartphone of their own accord. Accordingly, our study focused exclusively on the investigation of *active* non-compliance (i.e., participants noticed the beep but actively decided not to respond). In contrast, previous studies with their time-contingent designs did not differentiate between *active* and *passive* (i.e., participants did not notice a beep) non-compliance (Rogelberg et al., 2003). For example, in our study, if participants were doing sports in the morning and therefore did not use the smartphone, they did not receive and thus not miss any beep in this time interval. In contrast, in a study using a standard time-contingent ESM schedule, participants would have been notified to respond to a beep. Non-compliance could then either mean, that they did not notice the beep while doing sports in the morning or actively decided not to respond because, for example, they were enjoying their morning routine. The reason for deviating in the ESM design from previous literature was that we used smartphones not only to deliver beeps but also to collect mobile sensing data. If we had proactively notified participants, we would likely have altered their natural smartphone usage behaviors, which we included as features in our prediction models (van Berkel et al., 2019). Having this trade-off in mind, we decided to put emphasis on collecting naturally occurring (smartphone) behavior. In summary, our results should be interpreted depending on our study procedure, i.e., the times at which notifications were sent can be considered a pseudo-random sample of smartphone usage. For example, we found higher compliance rates on Sundays. However, it is important to keep in mind that fewer beeps than usual were sent on Sundays due to lower smartphone usage. Thus, people were less likely to receive ES notifications on Sundays because they used their smartphone less, but when they did use their smartphones, they were also more likely to respond. To allow more specific conditional interpretations of our results, we provide the distribution of the sent beeps depending on day, daytime, and day $\times$ daytime combination in our online material.

Second, when interpreting our results, we should keep in mind that the lack of some effects, e.g., for the *person* features, might be related to one major challenge of many empirical studies: self-selection or collider-stratification-bias (Bethlehem, 2010; Cinelli et al., 2022). Selection bias arises because our participants may not have entered our sample at random. Rather, the decision to participate in such a time-consuming, intensive longitudinal study is likely influenced by several factors, some of which might overlap with the factors investigated in our study. This impacts how we can interpret our results. This bias can be formalised by means of the directed acyclic graph (DAG) framework. We do not go into detail about the DAG framework at this point, but refer interested readers to Cinelli et al. (2022); Rohrer (2018), or Smith (2020). Nevertheless, we would like to briefly discuss the selection bias and possible consequences for the interpretation of our results in the light of the DAG framework to illustrate a possible scenario of how this bias might arise in ES and mobile sensing studies: On the one hand, someone with a demanding job might be less likely to join the study due to a lack of time. And if they do decide to participate, beep level compliance could be influenced by their job’s demands. This creates a situation where the job’s demands become a variable that affects both the decision to participate and their beep level compliance (Scollon et al., 2003; Stone et al., 2023). Thus, it constitutes a *confounder* variable. On the other hand, it should be considered that some features in our study, and probably especially stable person features, might have also affected the decision to take part in the study. For example, openness in previous research has been found to be related to the willingness to participate in surveys (Marcus & Schütz, 2005). If we then want to investigate how our features are related to beep level compliance, self-selection acts as a *collider* variable because it is affected by both our features (e.g., openness) and (unobserved) other factors (e.g., job demand). According to the DAG framework, to obtain unbiased estimates of the effect of our features on beep level compliance, we should then not condition our estimation upon a collider variable such as self-selecetion (Elwert & Winship, 2014). However, we automatically condition upon self-selection as we only consider data from persons taking part in our study, but not persons deciding against participating. Thus, conditioning on self-selection offers another possible explanation for the effect of our features on compliance at the beep level, at least for those features that also possibly affect self-selection (Cinelli et al., 2022). We speculate that this described constellation with confounders and colliders could especially apply to features of the *person* category and we might therefore not have found any associations with compliance. The issue of self-selection bias is common in psychological research, but probably especially so in studies like ours that involve intensive data collection. Therefore, future research should address the problem of person variables associated with self-selection in ES or mobile-sensing studies, for example, by contacting non-participants and learning about the "unknown." That is, by examining factors associated with the decision not to participate.

Third, our study and the associated ESM periods took place in July/August and October 2020, which might have led to some distortion of "normality" due to the ongoing COVID-19 pandemic. Accordingly, our results should be interpreted against the background of the COVID-19 pandemic, which may have led to changes in everyday behaviors and contextual characteristics (e.g., time spent at home). However, governmental restrictions in Germany were loosened during the time of data collection. For example, shopping restrictions were suspended, travel restrictions within Germany were loosened, and restaurants and daycares had started re-opening (as can be seen from the data collated by Steinmetz et al. , 2020). In line with this, we think that possible pandemic effects on our results are limited in scale. Nevertheless, future research should investigate whether our model generalizes outside of pandemic periods.

Lastly, as we wanted to include a broad range of mobile sensing-based behavioral and contextual features, we designed a research app running only on the Android operating system, as it allows more extensive access to third-party apps (Kreuter et al., 2020). However, only negligible to small differences in key personality traits have been found between users of the two most common smartphone operating systems, Android and iOS, which may be attributed to differences in the socio-demographic composition of the users (Götz et al., 2017; Keusch et al., 2020). Bearing this and the overall sample characteristics (e.g., size, age range, gender distribution) in mind, this rather supports the generalizability of our results (Götz et al., 2017).

## Conclusion

This study used a multitude of features of *person*, *behavior*, and *context* categories to predict compliance at the beep level in an ESM study. Based on a sample of 592 participants and more than 25,000 beeps, we used a combination of more than 400 features collected multi-methodologically via surveys, ES questionnaires, and mobile sensing. Compliance was successfully predicted at the beep level, with both linear and non-linear models investigated in our machine learning benchmark experiment. Using a large variety of person, behavior, and context features, we found indicators of a compliance bias in our ESM study. Our follow-up analyses revealed that study-related past behaviors were most informative in predicting compliance at the beep level, followed by physical context features related to participants’ mobility. In contrast, smartphone-mediated behaviors, temporal context, psychological context, and person characteristics played a negligible role in predicting compliance.

Our study has implications for both researchers applying ESM and those doing methodological research on ESM. With ESM being a widely used method across disciplines and smartphones being omnipresent and increasingly used in research, our study contributes a multi-method approach combining traditional and newer data-intensive collection methods to gain insight into compliance bias in ESM studies.

## Data Availability

The pre-processed feature data are available at https://osf.io/jw3bn/

## Footnotes

1 Within the context of missing data, variables that only aim to enhance the performance of statistical methods handling missing data even if they are not particularly relevant to the scientific hypotheses of interest, are called auxiliary variables (Collins et al., 2001)

2 preregistration is available at https://doi.org/10.23668/psycharchives.2901

3 https://osf.io/jw3bn/

4 For information on the PhoneStudy project see https://phonestudy.org/en/

5 https://developers.google.com/awareness/android-api/snapshot-api-overview

6 https://developers.google.com/awareness/android-api/fence-api-overview

7 https://developers.google.com/android/reference/com/google/android/gms/location/ActivityRecognitionApi

## Code Availability

The preprocessing and analysis code for the study is available at https://osf.io/jw3bn/
